# Chaperoning skin atrophy

**DOI:** 10.18632/oncotarget.26375

**Published:** 2018-11-23

**Authors:** Donald B. DeFranco

**Affiliations:** Associate Dean for Medical Student Research Professor and Vice Chair of Education, Department of Pharmacology and Chemical Biology, University of Pittsburgh School of Medicine, Pittsburgh, PA, USA

**Keywords:** glucocorticoid receptor, skin atrophy, FKBP51, Akt

The FK506-binding protein-51 (FKBP51) is a molecular chaperone best known for its interaction with nuclear receptors (NR), especially the glucocorticoid (GC) receptor (GR). Over the years, various functions have been attributed to FKBP51, including: control of intracellular localization of unliganded GR, inhibition of hormone induced GR nuclear translocation, and inhibition of GR hormone-binding capacity [1]. Yet none of these functions adequately explained another observation – that FKBP51 can exert dramatic and sometimes opposing effects on NR transcriptional activities – effects that are receptor- and target gene-specific. Recently, a new role for FKBP51 has been uncovered that can potentially explain its dynamic and diverse actions on NRs: control of specific kinases that phosphorylate the receptors. Regulation of NR activity by phosphorylation is well documented and is mediated by multiple kinases responding to various signals [2, 3]. Newly published evidence has shown that FKBP51 inhibits Akt phosphorylation, which can influence GR phosphorylation and function [4].

Although most molecular assays show FKBP51 to be a negative regulator of GR transcriptional activity, the relevance of these findings to therapeutic versus adverse effects of GCs has not been established, due in part to the lack of obvious developmental and health defects in mice with global FKBP51 knockout (KO) [1]. The lack of overt phenotypes in FKBP51 KOs has led to the hypothesis that FKBP51 may be required for stress-induced GR responses. For example, FKBP51 appears to impact GC contributions to depression and posttraumatic stress disorder [5].

Synthetic GCs are among the most effective and frequently prescribed anti-inflammatory drugs. However, they also are responsible for adverse metabolic and atrophic effects, including skin atrophy characterized by drastic hypoplasia and reduced skin barrier function [6]. As shown in this article, FKBP51 is one of several well characterized GR-target genes in skin [7]. As FKBP51 typically exerts negative effects on GR, it seems likely that in FKBP51 KO animals, GR activity in skin would be elevated and the anti-proliferative, atrophic effects of GCs would be enhanced.

In this work, Budunova and colleagues found that while the lack of FKBP51 did indeed result in increased AktSer473 and mTORSer2448 phosphorylation as expected, GR function was not affected in both mouse skin and human keratinocytes in *vitro.* Even more surprisingly,in FKBP51 KO animals, epidermis, dermal adipose and even CD43+ hair follicle stem cells were significantly protected against GC-induced atrophy. Because the Akt/ mTOR pathway negatively affects GR activity in skin [8], the authors concluded that Akt/mTOR activation, rather than altered GR activity, is the major mechanism for resistance of FKBP51 KO mice to the GC-induced skin atrophy. Overall, this work highlights complex, cell context-dependent regulation of GR function by FKBP51, and the novel role of Akt in this process.

Interestingly, the same group of authors recently identified another gene, REDD1 (regulated in development and DNA damage response 1) that plays an important role in GC skin atrophy [9]. REDD1 is a nutrient/energy sensor and an early stress-response gene activated by many cellular stresses, as well as GCs [10]. Even though both REDD1 and FKBP51 have pleiotropic functions, they play similar roles in negative regulation of mTOR/Akt signaling. While FKBP51 promotes Akt dephosphorylation at Ser473 [11], REDD1 promotes Akt dephosphorylation at Thr308 [12]. These interesting findings have translational potential as they build a foundation for development of safer GR-targeted therapies using GCs in combination with tissue protectors, such as small molecule inhibitors of the atrophogenes, FKBP51 and REDD1.
